# Stabilization of Immobilized Lipases by Intense Intramolecular Cross-Linking of Their Surfaces by Using Aldehyde-Dextran Polymers

**DOI:** 10.3390/ijms19020553

**Published:** 2018-02-12

**Authors:** Alejandro H. Orrego, Rohollah Ghobadi, Sonia Moreno-Perez, Adriana Jaime Mendoza, Gloria Fernandez-Lorente, Jose M. Guisan, Javier Rocha-Martin

**Affiliations:** 1 Department of Biocatalysis, Institute of Catalysis and Petrochemistry, Consejo Superior de Investigaciones Científicas (CSIC), 28049 Madrid, Spain; a.herrera@csic.es (A.H.O.); r.ghobadi60@gmail.com (R.G.); jama8507_@hotmail.com (A.J.M.); g.f.lorente@csic.es (G.F.-L.); 2 Department of Chemistry, Kharazmi University, 1417466191 Tehran, Iran; 3 Pharmacy and Biotechnology Department, School of Biomedical Sciences, Universidad Europea de Madrid, Villaviciosa de Odón, 28670 Madrid, Spain; sonia.moreno@universidadeuropea.es; 4 Departamento de Química, Universidad de Guadalajara, Guadalajara 44430, Jalisco, Mexico

**Keywords:** enzyme stabilization, lipase immobilization, stabilizing polymers, chemical amination, enzyme cross-linking, aldehyde–dextran

## Abstract

Immobilized enzymes have a very large region that is not in contact with the support surface and this region could be the target of new stabilization strategies. The chemical amination of these regions plus further cross-linking with aldehyde-dextran polymers is proposed here as a strategy to increase the stability of immobilized enzymes. Aldehyde-dextran is not able to react with single amino groups but it reacts very rapidly with polyaminated surfaces. Three lipases—from *Thermomyces lanuginosus* (TLL), *Rhizomucor miehiei* (RML), and *Candida antarctica* B (CALB)—were immobilized using interfacial adsorption on the hydrophobic octyl-Sepharose support, chemically aminated, and cross-linked. Catalytic activities remained higher than 70% with regard to unmodified conjugates. The increase in the amination degree of the lipases together with the increase in the density of aldehyde groups in the dextran-aldehyde polymer promoted a higher number of cross-links. The sodium dodecyl sulfate polyacrylamide gel electrophoresis (SDS-PAGE) analysis of those conjugates demonstrates the major role of the intramolecular cross-linking on the stabilization of the enzymes. The highest stabilization was achieved by the modified RML immobilized on octyl-Sepharose, which was 250-fold more stable than the unmodified conjugate. The TLL and the CALB were 40-fold and 4-fold more stable than the unmodified conjugate.

## 1. Introduction

Enzymes are nature’s catalysts for carrying out very complex chemical reactions. They have excellent catalytic properties such as high activity, selectivity, and specificity. However, their lack of stability does not allow for their implementation on an industrial scale. The stabilization of proteins allows for their use over longer periods of time and it also allows for their use under more drastic experimental conditions, such as higher temperatures or when using organic solvents [1]. Lipases display good stabilities for different reaction media [2,3,4]. However, it is critical to improve their stabilities in order to successfully provide economically attractive chemical processes.

Different immobilization protocols have been applied successfully to enhance enzyme properties such as stability, selectivity, and activity [5]. For example, lipases may be adsorbed on hydrophobic supports, becoming hyper-activated and highly stabilized (between 100- and 500-fold) [6]. However, the immobilization of proteins involves only a very small area of the protein surface; hence, a large region of the enzyme surface is not in contact with the support. This large region of the enzyme surface may be the object of new stabilization strategies. The promotion of intense cross-linking of amino groups from the enzyme surface using polyfunctional polymers is proposed here as an additional strategy for the stabilization of immobilized enzymes. In addition to Lys residues and the amino terminus existing on the enzyme surface, the number of reactive amino groups increases significantly with the transformation of a number of carboxyl groups (residues Asp and Glu) into amino groups. The chemical amination is carried out by the reaction of carboxyl groups, through a previous activation with soluble carbodiimide, and ethylenediamine. These new amino groups are more reactive than Lys residues because they present a lower p*K_a_* (around 9.3 for the new amino groups vs. 10.5 for the Lys residues) [7,8]. 

**Figure 1:**
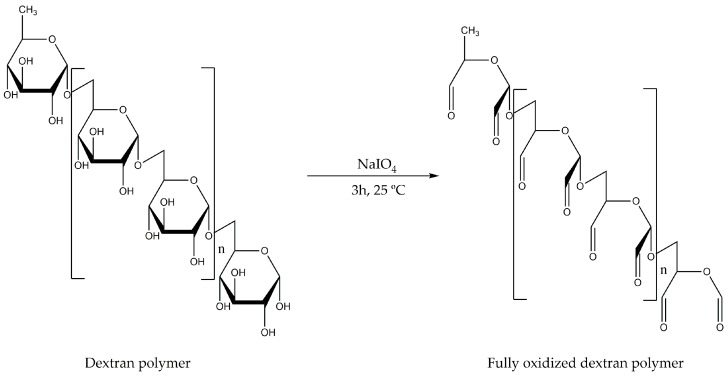
Periodate oxidation of dextran’s α-1,6-glucose residues.

Aldehyde-dextran was selected as a very adequate polyaldehyde polymer to cross-link the amino groups on the enzyme surfaces. In fact, aldehyde-dextran has already been used for cross-linking subunits of multimeric enzymes [9,10,11], decreasing enzyme desorption from supports [12,13], and preventing enzyme interactions with gas bubbles [14,15,16] or protein surface modifications for many kinds of proteins (e.g., oxidizing agents) [17]. Dextran is a polymer with a backbone composed of α-1,6-glycosidic linkages between glucose molecules, and aldehyde-dextran is obtained by the oxidation of dextran with periodate (Figure 1). The complete oxidation of the dextran polymer promotes the formation of two aldehyde groups via a glucose molecule (an extremely high concentration of aldehyde groups) [18]. Polyaldehyde-dextran is the main structure of freshly oxidized dextran. The reaction of single aldehyde groups and single amino groups forms very unstable Schiff´s bases and, hence, in the absence of stabilizing agents, these one-point attachments do not take place. However, incubation of this polymer at different pH values may yield other structures via the modification of vicinal aldehydes, such as aldo-enol, hydrated aldehydes, and hemiacetals [18,19]. These new cyclic structures are able to react with single amino groups on protein surfaces, leading to the formation of quite stable one-point attachments. For this reason, aldehyde-dextran has been used as an excellent cross-linking agent for the coating of enzyme surfaces and for intermolecular cross-linking [9,10,11,12,13,14,15,16,17].

We are here proposing an additional, novel aspect of freshly prepared aldehyde-dextran. Vicinal aldehyde groups coming from a glucose monomer could form interesting and stable two-point attachments with vicinal amino groups on a very highly aminated protein surface. These two-point attachments could become highly stabilizing cross-links because they are very short, and they could strongly reduce the relative mobility of each pair of cross-linked vicinal amino group. Each aldehyde-dextran molecule could form several stabilizing two-point stable attachments with amino groups on enzyme surfaces. The main property of these aldehyde groups is their high reactivity towards *N*-nucleophiles as amino groups, yielding very unstable one-point attachments but very stable two-point attachments when the attachment simultaneously involves two vicinal groups [20]. These two-point attachments may be favored because of the absence of steric hindrances for amino–aldehyde condensation.

In this paper, the reactivity of aldehyde groups of oxidized dextran was studied by immobilizing it on a Toyopearl-cysteine support [21]. A partially oxidized dextran-aldehyde was immobilized through Schiff’s base formation between the aldehyde groups of the dextran and the amino group of the cysteines. Once the partially oxidized dextran was covalently attached to the support, a second oxidation with sodium periodate was performed to obtain the activated form of dextran-Toyopearl (ADT). The model enzyme chosen for this experiment was penicillin G acylase (PGA) from *E. coli*. Finally, these polymers were used to stabilize three highly aminated immobilized lipases from *Themomyces lanuginosus* (TLL) [22], *Rhizomucor miehiei* (RML) [23], and *Candida antarctica* B (CALB) [24]. The lipases were adsorbed on octyl-Sepharose (OA). This well-known immobilization strategy allows the selective immobilization of lipase on hydrophobic supports due to its open form allowing their hyperactivation [6,12,25,26,27]. In addition, we took advantage of this feature to protect the lipase’s active center from possible undesirable modifications with aldehyde-dextran. 

## 2. Results and Discussion

### 2.1. Study of the Aldehyde-Dextran Reactivity

The aldehyde-dextran-Toyopearl (ADT) support was prepared to demonstrate that the aldehydes of an oxidized dextran polymer present the same chemical reactivity as the aldehyde groups of a glyoxyl-agarose support. Glyoxyl groups are short aliphatic aldehyde groups obtained from the oxidation of glyceryl groups. These aldehydes are stable at pH 10, which allows for immobilization onto the support through ε-amino groups from Lys residues [28]. This methodology, which achieved very high stabilization factors, has been applied for the immobilization-stabilization of many enzymes [29,30,31,32,33].

An epoxy-Toyopearl support was activated with cysteine, which has two nucleophilic groups (amino and thiol). This support is activated at pH 7.0 to leave the amino groups of the cysteine-Toyopearl support free. Thus, the support activation occurs only through the reaction between the thiol groups of the cysteine and the epoxy groups of the epoxy-Toyopearl support [21]. In this manner, the support containing only primary amino groups would react with the aldehyde groups from the 20% oxidized dextran polymer using the Schiff′s base mechanism in the next step of the activation. After reduction with borohydride, Schiff´s bases between amino and aldehyde groups are converted into secondary amino bonds. At this point, all the aldehyde groups of the support are converted to inert hydroxyl groups. To activate the support, a second oxidation would be necessary to obtain reactive aldehyde groups (ADT support).

**Figure 2:**
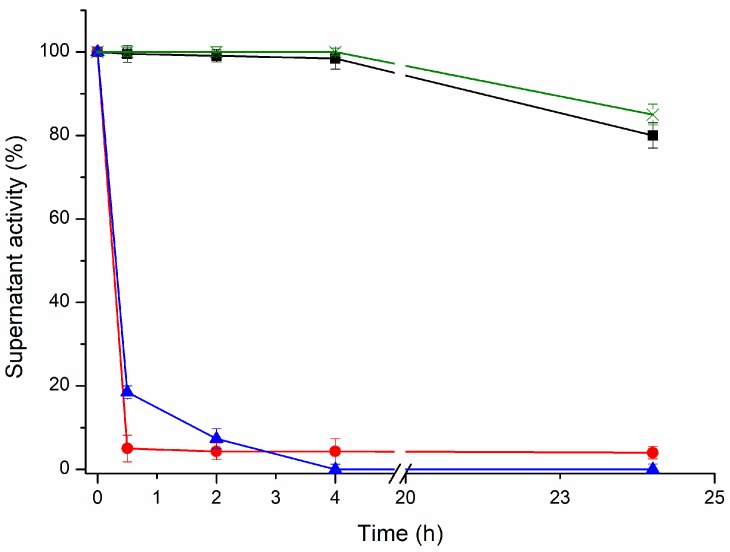
Time course immobilization of penicillin G acylase from *Escherichia coli* (PGA) on an aldehyde-dextran–Toyopearl (ADT) support with different buffers. Symbols: 50 mM sodium phosphate pH 8.5 (black squares); 50 mM sodium phosphate plus 30 mM dithiothreitol (DTT) pH 8.5 (red circles); 0.1 M sodium bicarbonate pH 10 (blue triangles); and soluble PGA control at pH 10 (green crosses).

PGA was selected as the model enzyme. It was immobilized on ADT support under different pH and buffer conditions (Figure 2). At pH 8.5 where only the amino terminal is non-protonated, only 2.5% of the PGA was immobilized on a freshly prepared ADT support after 4 h. A number of isolated aldehyde groups should compose the polymer and their reaction with single amino groups on the enzyme surface occurs via the formation of very unstable Schiff´s bases. However, in the presence of dithiothreitol (DTT), the one-point amino-aldehyde attachments become irreversible. This stabilizing effect of DTT on single Schiff’s bases has been previously reported [34]. This means that the amino terminus of the PGA is exposed to the medium but that it is unable to react by one-point attachment with aldehyde groups in the absence of stabilizers.

**Figure 3:**
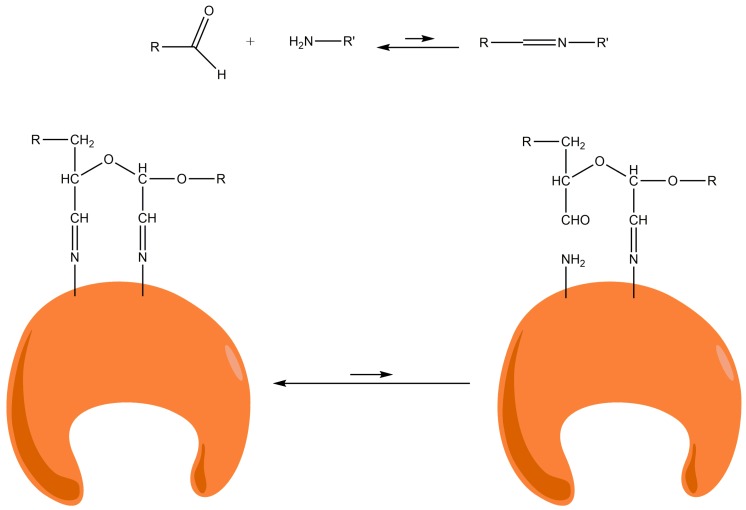
Schematic representation of a very stable attachment involving vicinal unstable Schiff’s bases.

At pH 10, 100% of the PGA was rapidly (4 h) and irreversibly immobilized. At this pH, the ε-NH_2_ groups of the Lys residues on the enzyme surface become partially non-protonated. Such immobilization seems to be due to the formation of stable two-point amino-aldehyde attachments. When the two-point attachment occurs between two amino groups of vicinal Lys and two vicinal aldehyde groups (e.g., produced on the same glucose monomer), there is an intramolecular stabilization of unstable bonds. When one attachment is broken, both the amino and aldehyde group remain very close and the local concentrations of both reagents are extremely high. In this way, the attachment is instantaneously restored and the two-point attachment becomes irreversible (Figure 3).

**Figure 4:**
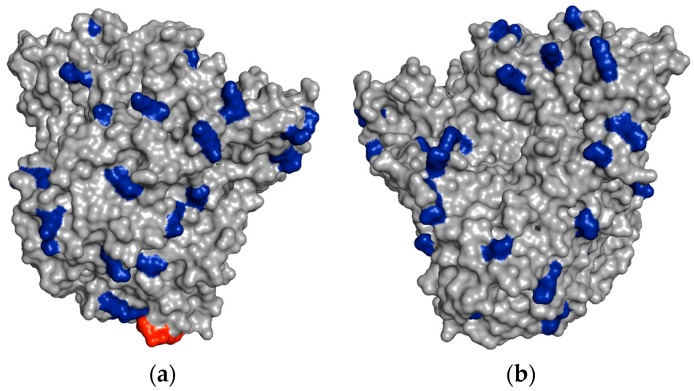
Vicinal Lys residues on the PGA surface. Lys residues are colored in blue and N-terminal is colored in red. (**a**) Front-side view of the PGA; (**b**) Back-side view of the PGA. The 3D structure was obtained from the Protein Data Bank (PDB) using PyMol v. 1.74. The PDB code for PGA is 1PNK.

In Figure 4, it is possible to see some regions of the PGA surface containing several vicinal Lys residues, which could explain the very rapid immobilization of PGA on aldehyde-dextran at pH 10.

**Figure 5:**
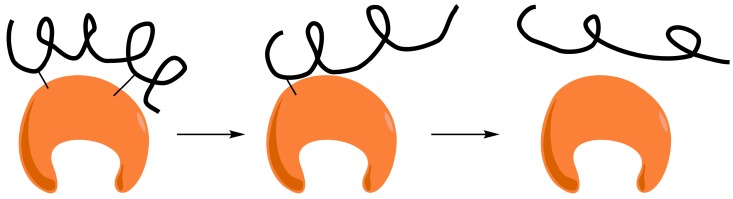
Schematic representation of the interaction process between two amino groups of non-vicinal Lys and two non-vicinal aldehyde groups placed at medium or long distances.

However, the stabilization of unstable Schiff′s bases by two-point attachment is not possible when two amino groups of non-vicinal Lys react with two non-vicinal aldehyde groups placed at a medium or long distance. Dextran is a very flexible polymer and there is no intramolecular stabilization due to two-point attachment. When one attachment is broken, the released amino and aldehyde groups separate very quickly to a distance where neither can interact (the aldehyde in the oxidized dextran moves very fast) and the attachment remains broken. Following this, the second attachment is broken again and irreversible immobilization is impossible (Figure 5).

The possible reaction mechanism between vicinal Lys and vicinal aldehyde groups could promote the interesting stabilization of enzymes via intramolecular cross-linking between highly aminated enzyme surfaces and freshly prepared aldehyde-dextran polymers.

In this way, polyaldehyde-dextran has three different reactivities:

1. Multipoint reaction with poly-aminated structures;
2. One-point attachment with single amino groups in the presence of Schiff’s base stabilizers;
3. One-point attachment between single amino groups and modified cyclic structures.

Bearing in mind all the properties of aldehyde-dextran, this polymer is almost ideal for the modification of protein surfaces:

- The density of aldehyde groups in the polymer is very high;
- There are no steric hindrances for the amino-aldehyde reaction;
- The reaction with a poly-aminated structure involves a number of multipoint covalent attachments in the absence of stabilizers.

Multipoint covalent attachments should occur between very close aldehyde groups and very close amino groups. That is, a number of stabilizing cross-links along the enzyme surface may be formed.

### 2.2. Cross-Linking of Lipases Adsorbed on Octyl-Sepharose Support

The main hypothesis of this work is that aldehyde-dextran is not only an excellent cross-linker agent but also a very good agent for the promotion of intense intramolecular cross-linking, leading to an increment in the stability of the proteins. To demonstrate this fact, the intramolecular cross-linking of three different lipases were studied as model enzymes. The three lipases were immobilized on a hydrophobic OA support that was not expected to suffer any modifications during the chemical amination of enzymes. Initially, two different variables were selected in order to reach different cross-linking intensities: the amination degree of the enzyme surface and the oxidation degree of a 6 kDa dextran-aldehyde. 

On the one hand, in order to obtain different amination degrees of the enzyme surface, the amount of 1-ethyl-3-[3-dimethylaminopropil]carbodiimide (EDAC) was gradually increased from 1 mM to 10 mM (for a modification of 50% to 100% of superficial carboxylic acids) [35]. Table 1 shows the number of Asp, Glu, and Lys from the different lipases used in this study. After the chemical modification of the carboxylic groups of Asp and Glu, the number of reactive amino groups available was increased depending on the amination degree. The exposed amino acid residues are more likely to react with aldehyde groups from the modified dextran.

**Figure 6:**
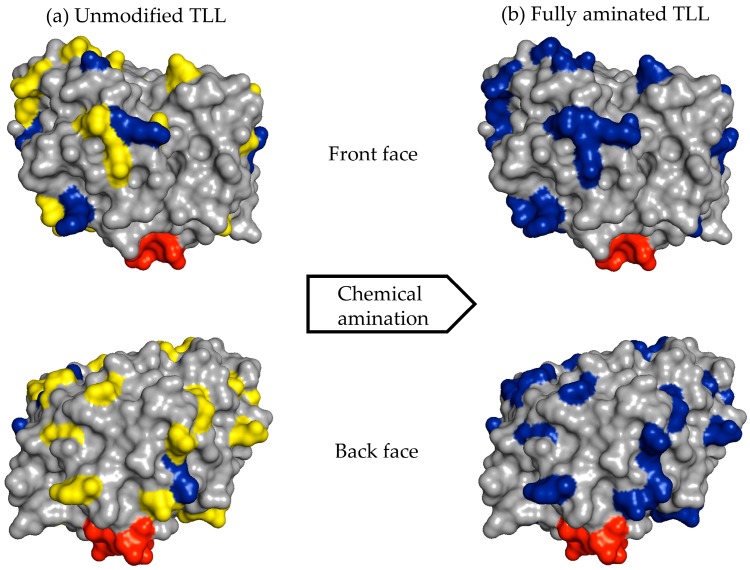
Unmodified and fully aminated surfaces of TLL. (**a**) Lid (red); Glu and Asp residues (yellow); and Lys residues (blue); (**b**) Lid (red); and Glu, Asp, and Lys residues (blue). The image was obtained using the PyMOL 1.74 program from the structure of the intact *Thermomyces lanuginosus* lipase (PDB code 1TIB).

For example, the unmodified TLL has six exposed Lys residues and one N-terminal that can react with the aldehyde groups of the modified dextran (Figure 6a). After full amination of the TLL, the number of exposed residues that had reactive amino groups could be increased to 30 (Figure 6b). In the other cases, the exposed amino groups were increased in CALB from 8 to 20 and in RML from 6 to 26 (Table 1).

On the other hand, the required percentage of aldehyde groups can be obtained by adding a certain amount of sodium periodate for the oxidation of the dextran polymer [20]. By controlling both parameters, different lipase preparations with different bond amounts between the enzyme and the dextran-aldehyde were obtained. 

In all cases, the recovered activities after the cross-linking process were over 70% of their initial catalytic activity (unmodified lipase-OA). The thermal stability of all the lipase preparations was studied by measuring the retained enzyme activity after 24 h using the unmodified lipases as a control. In the TLL preparations, the thermal stability was measured at pH 7.0 and 70 °C, while in the CALB and RML preparations the thermal stability was measured at pH 7.0 and 60 °C (Table 2). The use of soluble lipases as control was ignored since they were immediately denatured under those conditions.

A quick overview of the results shows a higher retained activity for most of the cross-linked preparations than the unmodified preparations. This means a higher thermal stability of the cross-linked conjugates than of the unmodified ones. Nevertheless, for TLL and RML, the modification with a full amination of the enzyme surface and an intramolecular cross-linking with a fully oxidized dextran led to the highest retained activity after the thermal inactivation process, from 7.5% to 48% and from 6.7% to 40% of retained activity after 24 h, respectively. Therefore, for these two lipases, a higher number of bonds between the enzyme and the dextran-aldehyde promotes an improvement in the thermal stability. For both enzymes, the other preparations with a smaller number of interactions between polymer and enzyme retained lower activities. Nonetheless, the results for CALB showed a different behavior. A fully aminated CALB surface and a fully oxidized dextran did not lead to an increment in the retained activity; this preparation retained 10.6% of its initial activity, whereas the unmodified CALB retained 10% after 24 h at 60 °C and pH 7.0. The highest increment in the retained activity for CALB was the preparation aminated with 1 mM of EDAC and cross-linked with a fully oxidized dextran, which showed a retained activity of 36.3% after the thermal inactivation process. In light of these results, the critical role of the cross-linking intensity is evident and reveals that it is necessary to study all the combinations to optimize the increment in the thermal stability of the enzymes.

### 2.3. Optimizing the Lipase Cross-Linking

Two more variables were studied to obtain an optimized coating of the surface of the lipases: dextran-aldehyde size and cross-linking time. The half-life of different preparations of lipases with different dextran sizes (from 1.5 kDa to 25 kDa) or cross-linking times (3 or 24 h) were studied under the optimal conditions of surface amination and oxidation degree of the dextran for RML and TLL.

For TLL, the optimal preparation presented a half-life of 36.7 h with a 24 h incubation with a dextran size of 25 kDa (Table 3). This optimal cross-linking reached a stabilization factor of 42- and 33.3-fold regarding the unmodified or aminated preparation, respectively (Table 3). In general, all the preparations increased the thermal stability of the immobilized and fully aminated TLL-OA by at least 15-fold. Moreover, no significant differences were found between the half-life times of the preparations, even when the preparations with the same dextran size but different cross-linking times were compared. This result is very interesting since in previous work reported by Rueda and co-workers, TLL immobilized on an agarose support and activated with octyl and glyoxyl groups showed a half-life of only 0.5 h, and the same biocatalyst when fully aminated showed a half-life of 2.25 h at 70 °C and pH 7.0 [37,38]. On the other hand, in a previous study conducted by our research group, PEGylation of aminated TLL adsorbed on an OA support led to obtaining a biocatalyst with a half-life of 20 h [39]. Here, our best biocatalyst had a half-life of 36.7 h under the same inactivation conditions (70 °C and pH 7.0). Therefore, the strategy applied in this work allows a greater improvement in the thermal stability of the TLL, keeping a significant percentage of its catalytic activity intact.

The optimal RML preparation was obtained with the same conditions of cross-linking time and dextran size (24 h and 25 kDa, respectively). This preparation achieved a half-life of 20.5 h, which is 256- and 128-fold more stable than the unmodified RML–OA and the fully aminated RML–OA, respectively (Table 3). As observed with the TLL preparations, which presented similar half-life times, RML preparations continued with the same tendency. Previous results reported by Rueda and co-workers showed a stabilization factor of the heterofunctional glyoxyl-octyl agarose that was 130-fold more stable than RML-OA at 50 °C and pH 7 [37]. Again, the applied strategy improved by 2-fold compared with the previous work.

All these results suggest that intramolecular cross-linking between aldehyde-dextran polymers and highly aminated enzyme surfaces is mainly responsible for the significant improvement in enzyme stability. The relative reaction rates of intermolecular (between lipase molecules) and intramolecular (only one lipase molecule) cross-linking are determined by the polymer concentration and chain length. Accordingly, at very low polymer concentrations or when using short-chain length polymers, intramolecular interactions dominate, yielding intense cross-linking of enzyme molecules [40]. Conversely, the use of long-chain dextran polymers promotes intermolecular cross-linking due to the covalent cross-linking of two or more of the enzyme’s molecules.

**Figure 7:**
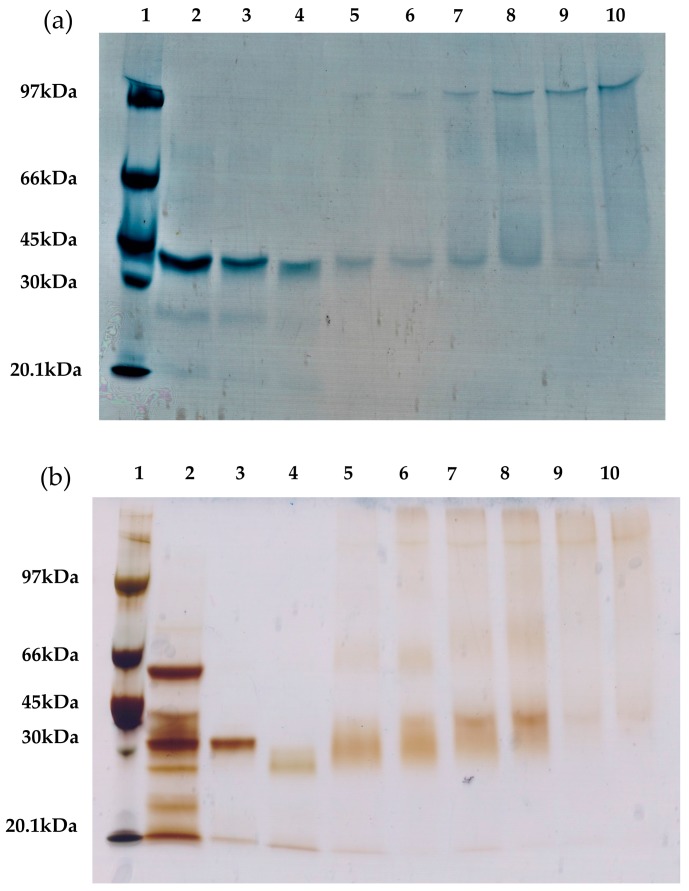
SDS-PAGE analysis of different lipase preparations of (**a**) TLL and (**b**) RML. Lanes: 1. Molecular weight markers; 2. soluble enzyme; 3. unmodified preparation; 4. aminated preparation; 5. cross-linked with 1.5 kDa Dx-CHO for 3 h; 6. cross-linked with 1.5 kDa Dx-CHO for 24 h; 7. cross-linked with 6 kDa Dx-CHO for 3 h; 8. cross-linked with 6 kDa Dx-CHO for 24 h; 9. cross-linked with 25 kDa Dx-CHO for 3 h; and 10. cross-linked with 25 kDa Dx-CHO for 24 h. (**a**) TLL: the soluble enzyme concentration was 0.35 mg/mL, and all the conjugates presented a concentration of 2 mg/g; (**b**) RML: the soluble enzyme concentration was 0.875 mg/mL, and all the conjugates presented a concentration of 5 mg/g.

To confirm that the intramolecular cross-linking is primarily responsible for the improved thermal stability of the lipases, different RML and TLL preparations were analyzed via sodium dodecyl sulfate polyacrylamide gel electrophoresis (SDS-PAGE) (Figure 7). The presence of a protein band of high molecular weight indicates intermolecular cross-linking (two or more molecules of the same enzyme are covalently attached). Conversely, the presence of protein bands with a similar size as that of the unmodified lipases indicate intramolecular cross-linking (only the dextran molecules are covalently attached to the enzyme molecules).

Comparing the different cross-linked preparations (Figure 7), the use of smaller dextran showed a higher amount of intramolecular cross-linked lipases. In contrast, the cross-linked preparations with the 25 kDa dextran polymer showed an increased presence of intermolecular cross-linking. On the one hand, this result confirms our previous assumption: the dextran polymers of smaller molecular weight promote intramolecular cross-links. On the other hand, cross-linking of RML or TLL preparations with a small aldehyde-dextran polymer (1.5 kDa) promotes very relevant stabilization, approximately 20-fold more stable than unmodified conjugates for both lipases. These stabilization factors are similar or significantly lower than the optimal cross-linked preparations with the 25 kDa dextran polymer. This fact proves the major contribution of the intramolecular cross-linking to the increment on the thermal stability of both immobilized lipases. Notwithstanding this, the cross-linking with a 25 kDa dextran polymer revealed that the intermolecular cross-linking also plays an important role in the stabilization of immobilized lipases. Likewise, it has been reported for RML that an increase in the dextran-aldehyde size (≥70 kDa) is the main factor responsible for reducing the desorption of RML molecules from the OA support since this molecular weight favored intense intermolecular and intramolecular cross-linking [12].

Finally, for both RML and TLL lipases, the optimal cross-linking was obtained after 24 h of cross-linking using the 25 kDa dextran polymer; the CALB was directly cross-linked under the same conditions. In this case, the cross-linking of the partially aminated CALB (1 mM EDAC) and the fully aminated CALB (10 mM EDAC) was tested. In Table 4 the results of the optimization of the cross-linking for the CALB-OA are summarized.

After the cross-linking with 25 kDa, both preparations (partial and fully aminated) presented higher thermal stability than the unmodified CALB-OA. What was more important was the increase in the thermal stability of the fully aminated and cross-linked preparation in view of the fully aminated CALB-OA, which was 14.3-fold more stable. On the contrary, the total amination of the unmodified CALB-OA destabilized the catalyst 3.4-fold (Table 4). Siddiqui and Cavicchioli modified the soluble CALB with oxidized 40 kDa dextran polymer [41]. The half-life of the modified CALB was only 1.4 h at 65 °C and pH 7.0. Our results are also an improvement from the previous ones published by Rueda and co-workers, who obtained an increase of 2.5-fold on the thermal stability using octyl-glyoxyl agarose as a support compared to CALB-OA at 70 °C and pH 7 [37].

## 3. Materials and Methods

### 3.1. Materials

The reagent 1-ethyl-3-[3-dimethylaminopropil]carbodiimide (EDAC) was purchased from Alfa Aesar (Karlsruhe, Germany). Ethylenediamine (EDA), sodium borohydride, sodium periodate, 2,4,6-trinitrobenzenesulfonic (TNBS) acid solution, Schiff’s reagent, all dextran polymers (dextran sizes ranged from 1.5 kDa to 40 kDa), p-nitrophenyl butyrate (pNPB), epichlorohydrin, dithiothreitol (DTT) and d,l-cysteine were acquired from Sigma (St. Louis, MO, USA). 6-Nitro-3-phenylacetamidobenzoic acid (NIPAB) was purchased from Fluorochem (Hadfield, UK). Octyl–Sepharose 4BCL (OA) was purchased from GE Healthcare (Uppsala, Sweden). Toyopearl HW65C was purchased from Tosoh Bioscience GmbH. (Griesheim, Germany). The enzymes *Candida antarctica* lipase B (CALB), *Thermomyces lanuginosus* lipase (TLL), and *Rhizomucor miehei* lipase (RML) were kindly donated by Novozymes (Bagsvard, Denmark). Penicillin G acylase (PGA) was kindly donated by Antibioticos S.A. (León, Spain). All other reagents were analytical or HPLC grade. Enzymatic assays were carried out on a V-730 spectrophotometer from JASCO Analitica Spain S.L. (Madrid, Spain).

### 3.2. Lipase Enzymatic Activity Assay

This assay was performed by measuring the increase in the absorbance at 348 nm (isosbestic point), which was produced by the released p-nitrophenol during hydrolysis of 0.4 mM pNPB in 25 mM sodium phosphate at pH 7.0 and 25 °C (the molar extinction coefficient (ε) is 5150 M^−1^ cm^−1^ under these conditions). The assay was performed continuously on a thermostatized and magnetic stirred cell. The reaction was initiated by the addition of 50 µL of lipase solution to 2.5 mL of substrate solution. One international unit (IU) of pNPB activity was defined as the amount of enzyme necessary to hydrolyze 1 µmol of pNPB per minute at pH 7.0 and 25 °C.

### 3.3. PGA Enzymatic Activity Assay

The enzymatic activity of PGA was deduced by measuring the increase in absorbance at 405 nm of 2.5 mL of 50 mM sodium phosphate buffer pH 7.0 and 0.15 mM of 6-nitro-3-phenylacetylamidobenzoic acid (NIPAB) at 25 °C. The measurement was performed continuously on a thermostatized and magnetic stirred cell (molar extinction coefficient (ε) is 9090 M^−1^ cm^−1^ in these pH and temperature conditions). One international unit (IU) was defined as the amount of enzyme required to hydrolyze 1 µmol of NIPAB per minute at pH 7.0 and 25 °C.

### 3.4. Dextran-Toyopearl Support Preparation

Ten grams of Toyopearl HW65C was resuspended in 30 mL of 0.8 M NaOH (0.96 g), 340 mg sodium borohydride and 11.4 mL acetone. The activation of the support started with the addition of 5.7 mL epichlorohydrin; after 2 h of reaction another 5.7 mL of epichlorohydrin was added, and the same amount of epichlorohydrin was added 4 h after the reaction. Finally, after 8 h at 25 °C under gentle stirring, the support was filtered and washed with an excess of distilled water. At this point, we had epoxide-Toyopearl. Ten grams of this epoxy-Toyopearl was incubated in 100 mL of 1 M of cysteine pH 7.0 solution for 72 h at 25 °C under gently stirring. After this time, the cysteine-Toyopearl support was filtered and washed with distilled water to remove the excess cysteine. 

Finally, 1 g of cysteine-Toyopearl was incubated in 10 mL of 0.1 M HEPES pH 8.5 and 30 mg/mL of aldehyde-dextran (25 kDa), which had previously been oxidized at 20% for 8 h at 25 °C under gentle stirring. Following this, solid sodium borohydride was added to a final concentration of 1 mg/mL and incubated for 30 min under the same conditions. After the reduction step, the support was filtered and washed both with 0.1 M sodium acetate pH 4.5 to remove the excess of borohydride and finally with distilled water. The presence of aldehydes or primary amine groups was qualitatively measured with Schiff’s reagent [42] or a TNBS assay kit [43].

### 3.5. Activation of Dextran-Toyopearl Support (ADT)

Before the immobilization of the proteins, it was necessary to activate the dextran–Toyopearl support using sodium periodate. One gram of support was incubated in 10 mL of distilled water and 0.8 g of solid sodium periodate. The oxidation of the remaining 80% of the glucose was carried out for 1.5 h at 25 °C under gentle stirring. Finally, the support was filtered and washed with an excess of distilled water. The result was the aldehyde-dextran-Toyopearl (ADT). The presence of aldehydes was confirmed by using Schiff’s reagent. The support was freshly prepared after each immobilization to avoid intramolecular aldol condensations of close aldehydes.

### 3.6. Immobilization of Penicillin G Acylase (PGA) on ADT

Three milligrams of PGA was immobilized onto 0.5 g of ADT in 50 mM of a specific buffer at room temperature under gentle stirring. The buffers consisted of sodium phosphate pH 8.5 with and without 30 mM of DTT or sodium bicarbonate at pH 10, 25% glycerin, and 100 mM phenylacetic acid. Samples of the suspension and supernatant were periodically withdrawn and its enzyme activity was measured during the immobilization process.

### 3.7. Lipase Immobilization

Two milligrams of TLL, 5 mg of RML, or 10 mg of *Candida antarctica* lipase B (CALB) in 10 mL of 25 mM sodium phosphate buffer pH 7.0 was added to 1 g of OA at 25 °C under gentle stirring. The lipases were immobilized by interfacial activation and the activity of the suspensions and supernatants were periodically measured using the pNPB assay. Finally, the immobilized lipase was filtered and washed with distilled water and with 25 mM sodium phosphate buffer pH 7.0.

### 3.8. Chemical Amination of Immobilized Lipases

One gram of conjugate was incubated in 1 M ethylenediamine pH 4.75 at 25 °C, containing different concentrations (10, 4, or 1 mM) of soluble carbodiimide 1-ethyl-3-[3-dimethylaminopropil] carbodiimide (EDAC) [44,45,46]. One-hundred percent of the carboxyl groups were modified with 10 mM carbodiimide, 80% were modified with 4 mM carbodiimide and 50% were modified with 1 mM carbodiimide [35,47]. After 1.5 h at room temperature under gentle stirring, the conjugate was filtered and washed with a 25 mM sodium phosphate buffer pH 7.0. The presence of additional amino groups was qualitatively confirmed by TNBS assay [43].

### 3.9. Dextran Aldehyde Coating

First, 3.33 g of dextran was dissolved in 100 mL distilled water. Different amounts of solid sodium periodate were added to get different percentages of aldehyde-dextran, taking into account that 2 moles of sodium periodate are needed to oxidize 1 mole of glucose (Table 1). The oxidation lasted 3 h at room temperature in the absence of light. Following this, the mixture was dialyzed against 50 volumes of distilled water to remove the released formic acid.

Table 5 shows the different oxidation parameters of the dextran polymer. A predefined amount of aldehyde-dextran was diluted in 25 mM phosphate pH 7.0 and added to 1 g of the conjugate and incubated for 16 h at 4 °C. Then, the pH was adjusted to 8.5 with 100 mM sodium bicarbonate, and solid sodium borohydride was added to a final concentration of 1 mg/mL. The reduction step lasted 30 min at room temperature. Finally, the conjugate was filtered and washed with 25 mM sodium phosphate pH 7.0 to remove all the excess of sodium borohydride.

### 3.10. Thermal-Stabilization Studies

The thermal inactivation assays were performed under different temperature conditions. The proportion of support and aqueous medium varied between 1/3 and 1/10 (*w*/*v*). Samples were withdrawn at different set times and residual activity was measured as described above. The remaining activity values were calculated as the ratio between the activity at a given time and the initial activity. Half-life times and inactivation constants were calculated as previously described [48].

### 3.11. SDS-PAGE Analysis

Sodium dodecyl sulphate polyacrylamide gel electrophoresis (SDS-PAGE) was performed as previously described [25]. The lipase conjugates were boiled in disruption buffer containing mercaptoethanol and SDS, thus releasing non-covalently bound protein from the support [9]. The protein concentration was the same for each sample. Samples were analyzed using 10% polyacrylamide gels, and proteins were detected by Coomassie blue staining (for TLL preparations) or silver staining (for RML preparations) [49].

## 4. Conclusions

**Figure 8:**
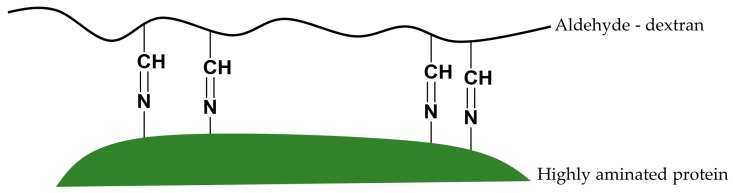
Intramolecular two-point cross-linking between freshly prepared aldehyde-dextran and highly aminated enzyme surfaces.

The thermal stabilities of fully aminated and cross-linked preparations of immobilized RML, TLL, and CALB with 25 kDa aldehyde-dextran polymer were increased by 250-, 40-, and 4-fold, respectively, with regard to the unmodified conjugates. The SDS-PAGE analysis of the cross-linked preparations confirmed the absence of intermolecular cross-linking when using low-size dextran (1.5 kDa). These preparations were also significantly stabilized. When the size of the dextran polymer was increased to 25 kDa, the preparations increased its stability no more than 2-fold. This fact suggests that the promotion of a number of intramolecular cross-links is the main stabilization cause [9]. Therefore, intramolecular two-point cross-linking between freshly prepared aldehyde-dextran and highly aminated enzyme surfaces (Figure 8) seems to play a major role in the additional stabilization of immobilized enzymes. These thermal stabilizations are on top of the stabilizations achieved by simple interfacial adsorption on hydrophobic supports (between 100- and 500-fold stabilization for one-point covalently immobilized conjugates) [6]. Hence, the resulting conjugates were dramatically stabilized for native lipases at around four or five orders of magnitude, and additionally they preserve more than 70% of the activity of native enzymes. These strategies for modifying large protein surfaces could also be applied to soluble enzymes (e.g., therapeutic enzymes) without needing a solid support.

## Figures and Tables

**Table 1:** Number of Lys, Glu, and Asp residues on TLL, CALB, and RML.

Residues	Lipase					
	TLL ^a^		RML ^b^		CALB ^c^	
	*Exposed*	*Buried*	*Exposed*	*Buried*	*Exposed*	*Buried*
Lys	6	1	5	2	7	2
Glu	10	2	10	3	3	1
Asp	13	6	10	5	9	5
N-terminal	1	-	1	-	1	-
NH_2_ groups after chemical amination	30	1	26	2	20	2

Protein surface accessibility was calculated using NetSurfP ver. 1.1 [36]. ^a^ TLL: *Thermomyces lanuginosus* lipase. ^b^ RML: *Rhizomucor miehiei* lipase. ^c^ CALB: *Candida antarctica* B lipase.

**Table 2:** Conserved activities (%) of the different immobilized lipase preparations after 24 h of thermal inactivation process.

Modification ^a^	Lipases		
	TLL ^b^	RML ^c^	CALB ^c^
Unmodified conjugate	7.5	6.7	10.1
50% amination plus 100% aldehyde-dextran	16.3	25.4	36.3
80% amination plus 100% aldehyde-dextran	23.7	33.2	24.4
100% amination plus 100% aldehyde-dextran	48.5	40.1	10.6
100% amination plus 60% aldehyde-dextran	16.1	32.8	20.6
100% amination plus 40% aldehyde-dextran	11.1	27.6	25.8
100% amination plus 20% aldehyde-dextran	7.5	20.3	-

The remaining activity is defined as the recovered activity on the solid support after the thermal inactivation process. ^a^ Amination and oxidation degree of aldehyde-dextran are as indicated. Aldehyde-dextran size was 6 kDa in all cases. ^b^ Remaining activity after 24 h of incubation at 70 °C and pH 7.0. ^c^ Remaining activity after 24 h of incubation at 60 °C and pH 7.0. All data are the mean value of three separate experiments where the error value was never higher than 5%.

**Table 3:** Half-life and stabilization factors of the different lipase cross-linked preparations.

Lipase	Parameter	Modification							
		Unmodified Conjugated	Totally Aminated Conjugated	Totally Aminated +Dx-CHO 1.5 kDa		Totally Aminated +Dx-CHO 6 kDa		Totally Aminated +Dx-CHO 25 kDa	
				3 h ^a^	24 h ^a^	3 h ^a^	24 h ^a^	3 h ^a^	24 h ^a^
TLL	t_1/2_ (h) ^b^	0.87	1.1	17.4	20.5	27.3	28.1	25.6	36.7
	Stabilization Factor ^c^	1	1.3	20.0	23.6	31.4	32.3	29.5	42.3
RML	t_1/2_ (h) ^b^	0.08	0.16	9.8	19	14.6	15.3	19.4	20.5
	Stabilization Factor ^c^	1	2	122.5	237.5	182.5	191.3	242.5	256.3

Half-life (in hours) and stabilization factors of different preparations of RML and TLL. The inactivation conditions were 70 °C and pH 7.0 for TLL conjugates and 60 °C and pH 7.0 for RML. ^a^ Cross-linking time is the incubation time of the aminated preparation together with the dextran aldehyde. ^b^ Half-life times were calculated as described in the Method’s section. ^c^ Stabilization factor is the relation of t_1/2_ of the modified preparation and the corresponding unmodified preparation. All data are the mean value of three separate experiments where the error value was never higher than 5%.

**Table 4:** Half-life and stabilization factor of optimal CALB preparations.

Conjugate	Half-Life ^a^	Stabilization Factor ^b^
CALB-OA	7.4	1
1 mM EDAC	7.1	0.96
10 mM EDAC	2.2	0.3
1 mM EDAC + 25 kDa dextran-aldehyde	24.6	3.3
10 mM EDAC + 25 kDa dextran-aldehyde	31.5	4.2

Half-life (in hours) and stabilization factor of different CALB preparation. The condition of the inactivation was 60 °C and pH 7.0. ^a^ Half-life times were calculated as described in the Method’s section. ^b^ Stabilization factor is the relation of t_1/2_ of the modified preparation and the unmodified preparation (CALB-OA). All data are the mean value of three separate experiments where the error value was never higher than 5%.

**Table 5:** Oxidation parameters of dextran-aldehyde.

Oxidation Degree of Dextran (%)	mmoles NaIO_4_/g Dextran ^a^	Amount of NaIO_4_ Added/g Dextran ^a^ (g)
20	2.22	0.47
40	4.44	0.95
60	6.67	1.43
100	11.1	2.38

^a^ Dextran sizes ranged from 1.5 kDa to 25 kDa. The size of the dextran polymer is irrelevant here since the amount of glucose monomers is the same in all dextran sizes.

## Abbreviations

TLL	*Thermomyces lanuginosus* lipase
RML	*Rhizomucor miehiei* lipase
CALB	*Candida antarctica* B lipase
PGA	Penicillin G acylase from *Escherichia coli*
OA	Octyl agarose
Dx-CHO	Aldehyde-dextran
EDAC	1-ethyl-3-[3-dimethylaminopropil]carbodiimide
pNPB	p-pitrophenolbutyrate
ADT	Aldehyde-dextran-Toyopearl
Asp	Aspartic acid
Glu	Glutamic acid
Lys	Lysine
Cys	Cysteine
DTT	Dithiothreitol
EDA	Ethylenediamine
TNBS	2,4,6-trinitrobenzenesulfonic
NIPAB	6-nitro-3-phenylacetamidobenzoic acid
